# Bilingual toddlers show increased attention capture by static faces compared to monolinguals

**DOI:** 10.1017/S136672892200092X

**Published:** 2023-01-20

**Authors:** Victoria L Mousley, Mairéad MacSweeney, Evelyne Mercure

**Affiliations:** 1 Institute of Cognitive Neuroscience, University College London, London, United Kingdom; 2 Deafness, Cognition and Language Research Centre, University College London, London, United Kingdom; 3 Birkbeck, University of London, London, United Kingdom; 4 Goldsmiths, University of London, London, United Kingdom

**Keywords:** bilingualism, toddlerhood, face processing, eye-tracking, visual attention

## Abstract

Bilingual infants rely differently than monolinguals on facial information, such as lip patterns, to differentiate their native languages. This may explain, at least in part, why young monolinguals and bilinguals show differences in social attention. For example, in the first year, bilinguals attend faster and more often to static faces over non-faces than do monolinguals (Mercure et al., 2018). However, the developmental trajectories of these differences are unknown. In this pre-registered study, data were collected from 15- to 18-month-old monolinguals (English) and bilinguals (English and another language) to test whether group differences in face-looking behaviour persist into the second year. We predicted that bilinguals would orient more rapidly and more often to static faces than monolinguals. Results supported the first but not the second hypothesis. This suggests that, even into the second year of life, toddlers’ rapid visual orientation to static social stimuli is sensitive to early language experience.

## Introduction

Primates’ visual attention is automatically captured by social stimuli such as conspecific faces (adult humans: Birmingham, Bischof & Kingstone, 2009; End & Gamer, 2017; Tomalski, Csibra & Johnson, 2009; apes: Kano & Tomonaga, 2011; Solyst & Buffalo, 2014). From just hours after birth, human infants look more rapidly to faces than to non-faces (Farroni, Johnson, Menon, Zulian, Faraguna & Csibra, 2005; Johnson, Dziurawiec, Ellis & Morton, 1991; Valenza, Simion, Cassia & Umiltà, 1996). There are strong theoretical frameworks suggesting that visual biases for social over non-social stimuli may be supported by face-specific subcortical pathways that are present at birth (Johnson, 2005, 2011; Johnson, Senju & Tomalski, 2015; Morton & Johnson, 1991; though see Simion, Macchi Cassia, Turati & Valenza, 2001). Morton and Johnson (1991) argued that the first face-specific subcortical mechanism, called Conspec, may be sensitive to the geometric properties of the human face in order to drive infants’ initial detection of faces in their environments. The second mechanism, named Conlearn, may support the infant’s eventual expertise in face recognition (Johnson, 2005, 2011; Johnson et al., 2015; Morton & Johnson, 1991). Infants’ universally rapid detection of human faces in the environment likely supports their swift social-communicative growth in early life. Early social biases likely provide infants a foundation on which to develop social cognitive skills, helping them learn to read facial expressions, identify gaze directions, and eventually elicit social interactions from caregivers (Lavelli & Fogel, 2002; Turati, Valenza, Leo & Simion, 2005).

Face processing influences social development, but early communicative experiences also shape the way children orient to and process social stimuli. Infants learning two spoken languages, for example, seem to capitalise on visual speech information available from people’s lips to perceive and discriminate their two native languages (Pons, Bosch & Lewkowicz, 2015; Weikum, Vouloumanos, Navarra, Soto-Faraco, Sebastián-Gallés & Werker, 2007). For example, Pons et al. (2015) found that four- and 12-month-old (but not eight-month-old) bilinguals spent longer looking to the mouths of dynamic, talking faces than monolinguals. In the current pre-registered study, we tested whether this pattern of increased attention to faces in bilingual compared to monolingual toddlers is also present in their visual scanning of static social and non-social stimuli. Specifically, we test whether bilingual 15- to 18-month-olds show heightened preferences for human faces compared to same-aged monolinguals, as measured as attention capture by and attention maintenance to faces over nonfaces in a static array. We expected that bilinguals would show faster first looks to faces over non-faces (i.e., attention capture) and look to faces for longer than non-faces (i.e., attention maintenance) compared to monolinguals.

**Figure 1:**
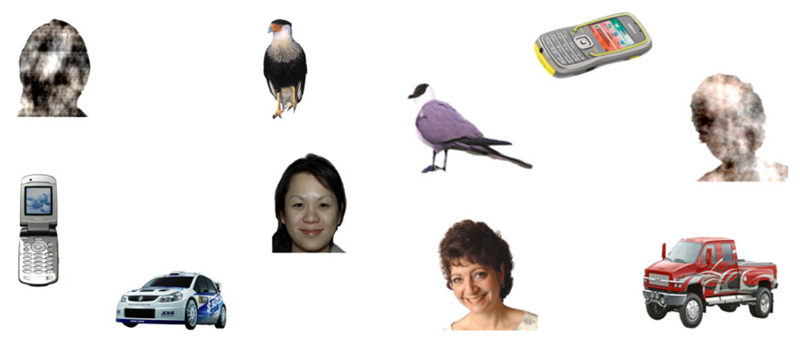
Example of face pop-out stimuli. *Note*. Two examples of face pop-out slides originally designed by Gliga et al. (2009). Each slide contained five areas of interest defined in MATLAB: face, noisy face, car, phone, and bird. The noisy face areas were created by randomising the phase spectra of the face on the slide while maintaining the outer face contour.

When presented with a static array containing one face and several non-face stimuli, infants are disproportionally likely to direct their first look to the face over non-face areas (Elsabbagh, Fernandes, Webb, Dawson, Charman, Johnson & BASIS team, 2013; Gliga, Elsabbagh, Andravizou & Johnson, 2009; Mercure, Quiroz, Goldberg, Bowden-Howl, Coulson, Gliga, Filippi, Bright, Johnson & MacSweeney, 2018; Telford, Fletcher-Watson, Gillespie-Smith, Pataky, Sparrow, Murray, O’Hare & Boardman, 2016). This ‘face pop-out’ effect was first measured by Gliga et al. (2009) by presenting infants with a static array containing one human face and four non-social stimuli (see Figure 1).

One of the non-social stimuli was a ‘noisy’ face stimulus, created by randomising the phase spectra of the image of the real face on the slide while maintaining the original outer face contour, with the amplitude and colour spectra remaining constant (Elsabbagh et al., 2013). Using this paradigm, Gliga et al. (2009) found that six-month-olds’ first fixations were faster to the face than non-face stimuli, suggesting faces have stronger ‘attention capture’ compared to non-face stimuli. The measure of attention capture relates to an individual’s propensity to identify social information in their environments (Gliga et al., 2009). Gliga et al. (2009) also found that infants showed stronger attention maintenance, measured as number of fixations, to the face over non-face areas while scanning the complex arrays (Gliga et al., 2009). Attention maintenance is related to an individual’s online monitoring of social information in their environment. Stronger attention capture by and attention maintenance to faces over nonfaces has since been replicated in different age groups, suggesting that infants’ increased attention to faces over non-faces is robust across six to 14 months of life (Elsabbagh et al., 2013; Gliga et al., 2009; Mercure et al., 2018). Face pop-out effects are also present in adulthood (Hershler & Hochstein, 2004). Attention capture by and maintenance to faces has also been shown in infants at risk of developing autism spectrum disorder (de Klerk, Gliga, Charman, Johnson & BASIS team, 2014; Elsabbagh et al., 2013) and in those born preterm (Telford et al., 2016). Thus, not only are face preferences present in primates (Kano & Tomonaga, 2011), human adults (End & Gamer, 2017), and consistent across human infancy, but they are also persistent across samples with wide developmental variability (de Klerk et al., 2014; Elsabbagh et al., 2013; Telford et al., 2016).

While most infants show strong biases to look to faces, some patterns of face processing are linked to variability in early communicative experiences. For example, infants learning two languages from birth (‘simultaneous bilinguals’) may rely more on facial cues than do monolinguals to bolster early language acquisition. Bilingual infants are exposed to two native languages. They therefore need to learn two distinct languages but with reduced amount of exposure to each compared to monolingual infants (Bosch & Sebastián-Gallés, 1997; Byers-Heinlein, Burns & Werker, 2010; Hoff, Core, Place, Rumiche, Señor & Parra, 2012; Werker, Yeung & Yoshida, 2012). Despite this, bilinguals and monolinguals develop remarkably similar language milestones (for review, see Byers-Heinlein & Lew-Williams, 2018). It seems likely that bilinguals must develop social communicative strategies to optimise their language learning processes. For example, research shows that bilinguals rely on lip patterns to help disambiguate their two native languages (Sebastián-Gallés, Albareda-Castellot, Weikum & Werker, 2012; Weikum et al., 2007). Whilst viewing talking faces, young bilinguals also tend to look more to the mouths than the eyes than do same-aged monolinguals (Pons et al., 2015; but see Morin-Lessard, Poulin-Dubois, Segalowitz & Byers-Heinlein, 2019). Bilingual infants look more to mouths of audio-visual face stimuli than monolinguals even when faces are not speaking but instead laughing or crying (Ayneto & Sebastián-Gallés, 2017). Taken together, this evidence suggests there may be differences between monolinguals and bilinguals’ processing of social information.

The bilingual effects on attention to faces described above were elicited by moving (Ayneto & Sebastián-Gallés, 2017) and/or speaking faces (Mercure, Kushnerenko, Goldberg, Bowden-Howl, Coulson, Johnson & MacSweeney, 2019; Pons et al., 2015; Sebastián-Gallés et al., 2012; Weikum et al., 2007). Research has begun to investigate whether bilingual effects may generalise to non-moving/non-talking faces. Two recent studies tested static face processing in bilingual and monolingual infants using the face pop-out paradigm. López Pérez, Tomalski, Radkowska, Ballieux, and Moore (2020) tested whether six- to seven-month-old monolingual and bilingual infants’ visual scanning behaviours while viewing static arrays predicted longitudinal language outcomes. They report no group differences in the pattern of return fixations on face pop-out slides between monolinguals and bilinguals. However, Mercure et al. (2018) reported language group differences in four- to 10-month-old monolingual and bilingual infants’ attention to static faces in two samples. Specifically, bilinguals showed stronger attention capture by and maintenance to static faces over non-faces than monolinguals (Mercure et al., 2018). When considered as ‘younger’ (four to eight months) and ‘older’ (seven to 10 months) samples, bilinguals’ increased attention capture by faces was driven by a borderline effect in the older infants (Mercure et al., 2018). There was no significant group difference in the younger sample’s (four to eight months) attention capture by faces (Mercure et al., 2018). The opposite was true for attention maintenance, such that younger but not older bilinguals showed more fixations to faces compared to monolinguals (Mercure et al., 2018). This pattern of results suggests that bilinguals return to look at static faces more than monolinguals in the first half of the first year, but that this effect may fade towards the second half of the first year, at which point bilinguals are faster to look to static faces than monolinguals (Mercure et al., 2018). Taken together, these results demonstrate that spoken-language bilingualism might impact infants’ strategies for viewing static faces over the first year of life, and further that these looking patterns may change over time.

Whether bilinguals’ increased attention to faces compared to monolinguals is a transitory phase, as suggested by the Mercure et al. (2018) study, or a group difference that persists into the second year of life is not clear. It could be that young bilinguals adopt strategies to support their achievement of goals specific to certain stages of development, but that early bilingualism is not related to longer-lasting differences in sensitivity to social information. For example, the group difference could reflect periods of intense phonological learning in the first year of life (perceptual attunement; for review, see Werker, 2018), where mouth movements are particularly important and informative. Bilinguals’ increased attention to faces in the first year may reflect increased effort during this phonological learning phase. It seems likely that young bilinguals may orient quickly to and remain looking at static faces because, in the event of speech, lip patterns are useful to them. This may or may not continue to drive group differences between monolinguals and bilinguals into the second year of life. The present study tests this question by comparing 15- to 18-month-old monolingual and bilingual toddlers’ attention capture by and maintenance to static faces over non-faces.

## Pre-registered hypotheses (https://osf.io/92tsa)

Bilingual toddlers were predicted to show increased attention to faces compared to monolinguals. Specifically, the pre-registered hypotheses were that:

1. Bilinguals would show stronger attention capture, defined as faster first looks, by faces over non-faces than monolinguals.
2. Bilinguals would show stronger attention maintenance, defined as a higher number of fixations, to faces over non-faces than monolinguals.

### Method

Parents of monolingual and bilingual toddlers were recruited from the Birkbeck Babylab database of volunteers to participate in a larger bilingualism project which included research on speech perception, attention to static and dynamic faces, cognitive control, visual attention, and parent-child interaction. The results from the attention to static faces task are reported here. Volunteers for the Babylab database were recruited from media, newsletters, and science communication events. All participants’ travel expenses were reimbursed, and families were offered a baby T-shirt and certificate of participation after their sessions. The study was approved by the UCL and Birkbeck Research Ethics Committees.

The lab visit included an eye-tracking battery, parent-child interaction protocol, and behavioural measures. During eyetracking, toddlers sat on their parent’s lap in a dimly lit, featureless room approximately 65 cm away from the Tobii TX300 presentation screen. Eye-tracking tasks included face pop-out and four other tasks not reported here. Blocks were randomly interleaved to maintain toddlers’ attention. Before the tasks began, the toddler’s gaze was calibrated with colourful, swirling animations using a five-point calibration routine. Each toddler’s gaze and behaviour were monitored throughout the study via webcam mounted on top of the presentation screen. The experimenter occasionally shook a rattle behind the screen to attract the toddler’s attention. After eye-tracking, participants completed behavioural measures relevant to other research questions in the wider project. Language interviews were administered to bilingual families. The entire protocol required between one-and-a-half to three hours per toddler including breaks.

### Participants

Valid data from *N* = 71, 15- to 18-month-old monolingual and bilingual toddlers were collected on the face pop-out task. Of these, *n* = 58 valid datasets (*n* = 32 monolinguals) were collected before the onset of the COVID-19 pandemic. In line with the preregistered plan (https://osf.io/92tsa), a further *n* = 13 valid datasets were collected 15 months into the COVID-19 pandemic once testing had resumed. These toddlers’ (*n* = 13) early lives took place during the pandemic amongst lockdowns, social distancing, and whilst caregivers and adults in their environment were required to wear facial masks. Fascinating research is beginning to examine whether/how experience with masked faces during the first two years of life is linked to unique social-communicative developments (see Carnevali, Gui, Jones & Farroni, 2022). However, because the present study was not designed to test effects of the pandemic on face processing, the post-pandemic participants were excluded from the present analysis. For the sake of full transparency, analysis including all participants can be found on the OSF (https://osf.io/78w4p/; visualisation of pre- and post-pandemic data in Figure S4).

Thirteen pre-pandemic toddlers (*n* = 7 monolinguals, *n* = 6 bilinguals) were excluded for having fewer than six valid trials, resulting in a total sample size of *N* = 58. Parents of all participants reported their child did not have a history of hearing or vision problems, seizures, or any serious mental or physical conditions. Toddlers were on average *M* = 16.38 months (*SD* = 1.12, range = 15.07 to 18.98 months). The groups did not differ in their ages (*t*(54.67) = -1.65, *p* = .105), their familial income (*t*(43.76) = 1.80, *p* = .078), the average level of education attained by mothers (*t*(48.68) = 1.15, *p* = .254) nor fathers (*t*(49.61) = 1.20, *p* =.236), the number of participants attending nursery (monolingual: *n* = 10, bilingual: *n* = 11), nor, for the children who did attend nursery, in the number of hours spent in nursery per week (*t*(45.30) = 1.02, *p* = .314). Monolinguals (*n* = 32; 20 boys) were only exposed to English. At the time of recruitment, parents of toddlers included in the bilingual group reported that their child was exposed to English as well as another language between 20 and 80% of the time. The existing developmental literature varies widely in the threshold of language exposure used to classify children as bilingual (Byers-Heinlein, 2015). In this case, the criteria of at least 20% exposure to a second language was chosen because this threshold has been used in previous research on visual attention to faces in infancy (e.g., Hillairet de Boisferon, Hansen Tift, Minar & Lewkowicz, 2016). The range of bilinguals’ 20 to 80% exposure to a second language was intentionally large given that the theoretical question asked in this study was whether bilingualism, across the wide variability in bilinguals’ early language experiences, was related to differences in attention to static faces over non-faces. Any effects that should emerge would therefore be more generalisable to a heterogenous group of children than would have been the case if a narrower definition of bilingualism (e.g., 50% exposure to each language) had been chosen.

A MacArthur-Bates Communicative Development Inventory was sent to all families within one week of their visit to the lab (Fenson, Marchman, Thal, Dale & Reznick, 2007). Monolinguals had an average receptive vocabulary of *M* = 248 words and productive vocabulary of *M* = 46 words. For bilinguals, average receptive vocabulary in English was *M* = 121 words compared to average receptive vocabulary in their second language of *M* = 128 words. Productive vocabulary amongst bilinguals was, on average, *M* = 38 words in English and *M* = 26 words in their second language. While the pre-registered hypotheses were not concerned with toddlers’ vocabulary sizes, we used these averages as a sense check that bilingual toddlers demonstrated similar proficiency in English and their second language.

The Language Exposure Questionnaire (‘LEQ’), a commonly used tool with bilingual families, was used to quantify percentage of bilinguals’ language exposure (Bosch & Sebastián-Gallés, 1997; Carbajal & Peperkamp, 2020; Kalashnikova, Pejovic & Carreiras, 2020; Potter, Fourakis, Morin-Lessard, Byers-Heinlein & Lew-Williams, 2019). The LEQ is a structured interview that quantifies a child’s early exposure to two languages. Caregivers provide information about their own language backgrounds. They are then talked through a typical day in the child’s life for each day of the week and asked to provide details about the language(s) spoken directly to the child in their various environments (i.e., home, nursery, during typical weekday and weekend activities). From this, the number of hours a child hears their two native languages can be calculated and a percentage score of exposure to each language is produced. For the present study, the percentage of exposure to each language was only used to confirm inclusion in the bilingual group. On average, bilingual toddlers (*n* = 26, 14 boys) had 58.91% exposure to English and 41.09% exposure to a non-English language. The non-English languages bilingual toddlers were learning French (*n* = 6), Spanish (*n* = 3), Italian (*n* = 2), Danish (*n* = 2), Mandarin (*n* = 2), Czech, Greek, Hungarian, Hebrew, Polish, Russian, Swedish, Tamil, Twi, Welsh, and Yoruba (all *n* = 1).

### Procedure and stimuli

The face pop-out task has been widely implemented in developmental research to measure attention directed to static face over static non-face stimuli (i.e., car, phone, noisy face, and bird) (Elsabbagh et al., 2013; Gliga et al., 2009; Mercure et al., 2018). Eight different slides were presented for 10 seconds each. Each slide included five colour images belonging to five categories: faces, noisy faces, birds, cars, and phones (see Figure 1). Each slide was presented once, and the areas of interest were presented in random locations on each slide. Images were all of comparable size (approximately 5.2° x 7.3°) and presented at an equal distance from the centre of the screen. Differences in colour and luminosity were minimised. Faces all had a direct gaze and happy expression. There were five female faces and three male faces of different ethnicities. Noisy faces were created from each face by randomising the phase spectra while maintaining the original outer face contour, with the amplitude and colour spectra remaining constant. For more details about the stimuli, see Elsabbagh et al. (2013).

### Data pre-processing and analysis plan

Continuous raw eye-tracking data were resampled to 60Hz, offscreen gaze was marked as missing gaze data, X and Y coordinates were averaged when data from both eyes were present, and data from one eye were used when data from one eye were missing. As in Portugal, Viktorsson, Taylor, Mason, Tammimies, Ronald, and Falck-Ytter (2022), five AOIs corresponding to the five stimulus categories (i.e., faces, noisy faces, birds, phones, and cars) were defined in MATLAB. Raw data were assigned to each AOI with logical vector codes of gaze samples either within (1) or outside (0) each AOI. AOI vectors were interpolated to fill gaps of missing data shorter than 200ms. Any runs of samples in an AOI vector that was less than 50ms were recoded to zero to ensure a minimum of 50ms of gaze data was accumulated in each AOI for the look to be computed.

Face pop-out trials were excluded when participants looked at the AOIs for less than one second in total. Thirty-two out of all possible 464 trials were excluded (monolinguals = 15 trials, bilinguals = 17 trials). Fourteen participants were excluded for analyses for completing four or fewer valid trials of the eight total face pop-out presented as per previous research on this task (Elsabbagh et al., 2013; Mercure et al., 2018). There were an equal number of monolinguals and bilinguals excluded for this reason (*n* = 7 each), and there were no group differences in the amount of time children looked to the screen overall.

After excluding trials and participants according to the exclusion criteria, fixation latency and fixation count were averaged across all participants’ valid face pop-out trials to normalise for the number of trials completed. Fixation latency (attention capture) was defined as the time difference between the trial onset and the first saccade to the face AOI. Fixation count (attention maintenance) was defined as the number of fixations to the face AOI. These measures were chosen specifically to test the research questions about whether bilingualism relates toddlers’ propensity to search for social information (attention capture) and their online monitoring of it (attention maintenance). As we did not have any specific hypotheses regarding group differences in attention to birds, cars, phones, or noisy faces, these categories were averaged to create a ‘non-face’ stimulus category (Mercure et al., 2018). However, in line with Mercure et al. (2018), any significant AOI effect was followed by planned, pairwise t-tests (Bonferroni-corrected) between the face and each non-face AOI to test whether any potential AOI effect was driven by all of the non-face areas and not by one in particular (https://osf.io/92tsa). Each pairwise comparison was expected to be statistically significant, as in Mercure et al. (2018), such that the faces would attract faster saccades and a higher number of fixations than each non-face stimulus category (for full analysis script, see https://osf.io/78w4p/).

The pre-registered analysis plan consisted of two 2 (AOI: face vs non-face) x 2 (Group: monolingual vs bilingual) mixed effects ANOVAs on two outcomes of interest: attention capture and attention maintenance (https://osf.io/92tsa). A main effect of AOI for both outcomes was expected, indicating an overall preference for face over non-face stimuli in all toddlers (Gliga et al., 2009; Mercure et al., 2018). Hypotheses regarding the impact of language experiences were tested by the AOI x Group interactions of each ANOVA. For the first hypothesis of bilinguals’ increased attention capture, a significant AOI x Group interaction was expected such that bilinguals would show stronger attention capture by faces over non-faces than monolinguals. The second hypothesis was that there would be a significant AOI x Group interaction for attention maintenance, such that bilinguals would show stronger attention maintenance, or higher number of fixations, to faces over non-faces than monolinguals.

## Results

### Tests of pre-registered hypotheses

**Figure 2:**
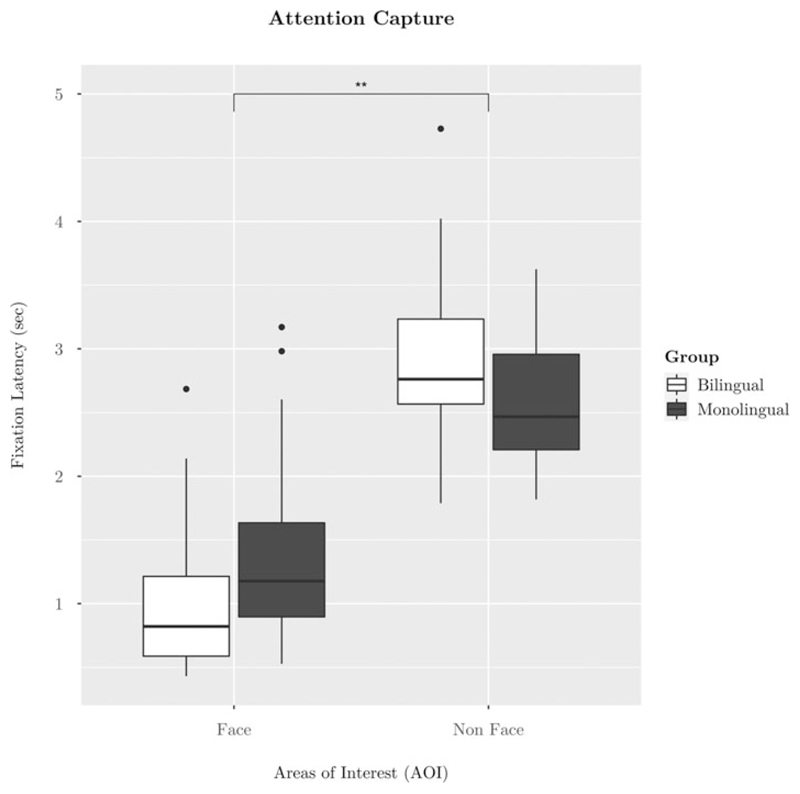
Boxplot of significant AOI x Group interaction on the outcome of attention capture (Hypothesis 1). Note. Bilinguals and monolinguals differed in their attention capture by faces and non-faces, such that bilinguals were faster to look to faces and slower to look to non-faces than monolinguals. Bilinguals are represented in white, and monolinguals are represented in grey. Fixation latency is calculated as the time between onset of the face pop-out slide and toddlers’ first fixation to the area of interest (face or nonface). The non-face values were calculated within-participant as an average of toddlers’ fixation latencies to the four non-face areas (i.e., noisy face, car, phone, and bird). The error bars represent standard error.

The first hypothesis of increased attention capture by faces in bilinguals than monolinguals was tested with a 2 (AOI: face vs non-face) x 2 (Group: monolingual vs bilingual) mixed effects ANOVA on fixation latency (https://osf.io/92tsa). As expected, there was a main effect of AOI (*F*(1,56) = 181.72, *p* < 0.001, $\eta_{\text{p}}^{2}=0.76$), such that all toddlers looked more rapidly to the face than the non-face areas (see Table 1). Each planned, pairwise t-test (Bonferroni-corrected) between attention capture by the face area and each non-face area (i.e., noisy face, car, phone, and bird) was significant (*p* < 0.001), indicating the effect of AOI was systematic across all non-face stimuli (see Table 1). There was no main effect of group. The hypothesized interaction between AOI x Group was also significant (*F*(1,56) = 7.86, *p* =.007, $\eta_{\text{p}}^{2}=0.12$) (see Figure 2). A t-test revealed a significant group difference in attention capture by faces, such that bilinguals looked more quickly to faces than did monolinguals (*t*(55.91) = 2.02, *p* =.049, *d* = -0.52; see Figure 2 and Table 1). There was also a significant effect of bilinguals’ reduced attention capture by non-face areas compared to monolinguals (*t*(46.73) = -2.12, *p* =.039, *d* = 0.57) (see Figure 2 and Table 1). While the attention capture variable was not normally distributed, when data were log transformed and analyses were re-run, the result remained the same.

**Figure 3:**
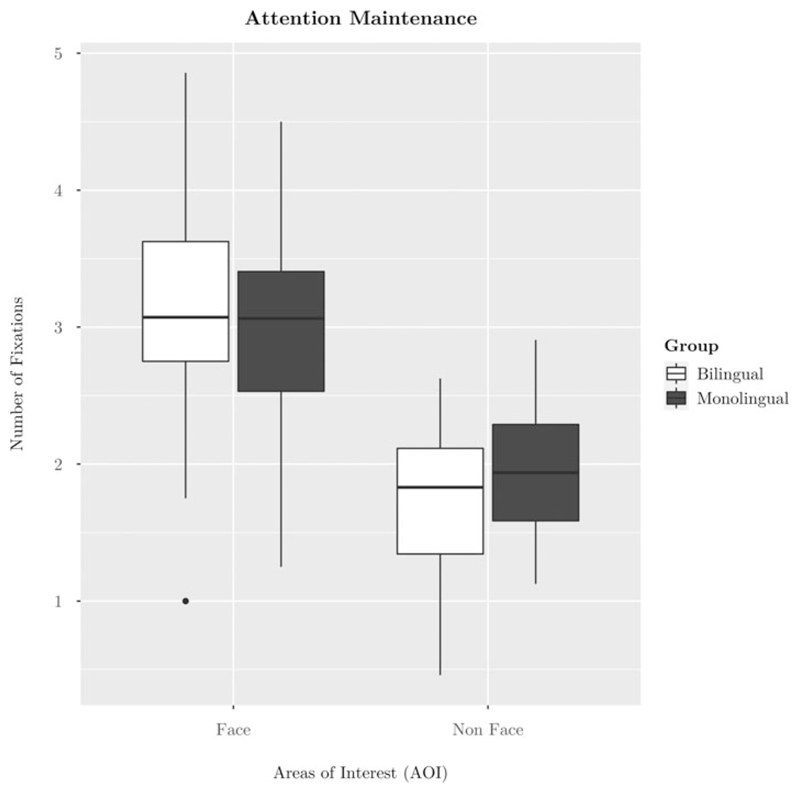
Boxplot of non-significant AOI x Group interaction on the outcome of attention maintenance (Hypothesis 2). *Note*. There was a main effect of AOI but not of group. The interaction of AOI x Group was not significant. Bilinguals are represented in white, and monolinguals are represented in grey. Fixation maintenance is calculated as the average number of return fixations to the areas of interest (face or non-face) that each participant made per completed trial. The non-face values were calculated within-participant as an average of toddlers’ fixations to the four non-face areas (i.e., noisy face, car, phone, and bird). The error bars represent standard error.

The second hypothesis of bilinguals’ increased attention maintenance to faces than monolinguals was tested with an identical structure to that of attention capture: a 2 (AOI: face vs non-face) x 2 (Group: monolingual vs bilingual) mixed effects ANOVA on the outcome of number of fixations. As expected, the results revealed a significant main effect of AOI (*F*(1,56) = 99.97, *p* < 0.001, $\eta_{\text{p}}^{2}=0.64$) such that all toddlers fixated to the face area more frequently than they did the non-face areas (see Figure 3 and Table 1). There was no main effect of group. Planned t-tests comparing number of fixations to the face and each non-face region revealed a significantly higher number of fixations to the face than each non-face area (all *p* < 0.001) (see Table 1). However, the hypothesized interaction of AOI x Group, which would indicate that bilinguals maintained their attention more to the face over non-face areas compared to monolinguals, was not significant (*F*(1,56) = 2.02, *p* = .161, $\eta_{\text{p}}^{2}=0.04$) (see Figure 3 and Table 1).

### Exploratory results

Several exploratory analyses were conducted to examine group differences in looking to faces versus non-faces. A 2 (AOI: face vs non-face) x 2 (Group: monolingual vs bilingual) mixed effects ANOVA was conducted on the outcome of overall look duration. There was a significant main effect of AOI (*F*(1,72) = 15.45, *p* < .001, $\eta_{\text{p}}^{2}=0.18$) such that all toddlers looked longer to face areas than non-face areas (see Figure S5). There was no main effect of group nor of AOI x Group interaction. A second 2 (AOI: face vs non-face) x 2 (Group: monolingual vs bilingual) mixed effects ANOVA was conducted on the outcome of proportion of looking to face of all looking to the screen. Again, there was a significant main effect of AOI (*F*(1,72) = 14.17, *p* < .001, $\eta_{\text{p}}^{2}=0.17$) such that all toddlers looked proportionately more to faces than elsewhere on the screen (see Figure S6). There was no main effect of group nor of AOI x Group interaction. Finally, a t-test was used to determine whether the groups differed in the number of areas of interest visited. There was no significant difference (see Figure S7).

## Discussion

The present study suggests that visual orientation mechanisms for social stimuli, generally considered robust across age and developmental variability, are also sensitive to early language experience. Fifteen to 18-month-old bilinguals were faster to look to faces and slower to look to non-faces than same-aged monolinguals. There were no group differences in the number of times toddlers fixated to the face over non-face areas. These findings contribute to the mounting evidence that face processing skills are linked to early language experience and that this experience differentially impacts various stages in development.

## Attention capture

As predicted in the first pre-registered hypothesis, bilinguals showed stronger attention capture by faces over non-faces than did monolinguals (see Figure 2). A paired t-test revealed bilinguals were significantly faster to look to the face than were monolinguals. This replicates a past study with younger infants that also reported stronger attention capture by faces in bilinguals compared to monolinguals between the ages of four and 10 months (Mercure et al., 2018). When considered as two separate samples (four to eight and seven to 10 months), only Mercure et al.’s (2018) older sample showed a borderline effect of language group on speed of first looks to the face over non-face areas (Mercure et al., 2018). The younger monolingual and bilingual infants did not show differences in their speed of first looks to the face compared to the non-face areas (Mercure et al., 2018). It may be that, with increased language experience, bilingual toddlers learn to orient quickly to faces to seek visual articulation information that boosts their perception and discrimination of two native languages (Pons et al., 2015; Sebastián-Gallés et al., 2012; Weikum et al., 2007). Research with adults does suggest that visual search behaviours are shaped by rewards associated with the targets (Kristjánsson & Campana, 2010) and that selective attention is influenced by the significance objects have gained through experience (Chelazzi, Perlato, Santandrea & Della Libera, 2013). Indeed, the emergence of bilinguals’ stronger attention capture by faces compared to monolinguals progresses developmentally. The difference between monolinguals’ and bilinguals’ attention capture by faces is not robust at four to eight months of age; but, by seven- to 10-months, the effect is apparent (Mercure et al., 2018). As demonstrated in the current study, the group difference is well-established by 15- to 18-months. The developmental pattern described here aligns with the interpretation that, through experience, bilinguals learn the value of face information to access communicative signals that optimise dual language learning.

No predictions were made regarding differences between monolinguals’ and bilinguals’ looking patterns to non-face stimuli. Pairwise contrasts followed the significant interaction showed above, such that bilinguals took longer than monolinguals to attend to the non-face areas of the static array (Figure 2). This is a novel finding. Past research with younger infants showed no group differences in speed of first looks to non-face areas (Mercure et al., 2018). In the present study, there were no group differences in the average duration of the first look to the face. It does not appear to be the case that bilinguals’ slower looks to the non-face areas were driven by longer first looks to the face area than monolinguals. Further, there were no differences between monolinguals’ and bilinguals’ overall duration of looking to the face area over non-face areas (see Figure S5) nor in the proportion of looking time to the face area over all looking time to the screen (see Figure S6). It may simply be that bilinguals’ slower looking to the non-face areas than monolinguals are a consequence of bilinguals’ strong attention capture by the face areas. If there were no face on the slide, we would not predict group differences in monolingual and bilingual toddlers’ speed of first looks to the non-face areas. This aligns with recent findings by López Pérez et al. (2020), who examined six- to seven-month-old monolingual and bilingual infants’ scanning of complex arrays containing only non-social areas and reported no group differences (López Pérez et al., 2020).

Some suggest that early exposure to two languages may be linked to rapid visual shifting to novel stimuli (Comishen, Bialystok & Adler, 2019; Dal Ben, Killam, Pour Illiaei & Byers-Heinlein, 2022; Kovács & Mehler, 2009; but see Kalashnikova et al., 2020; Tsui & Fennell, 2019). For example, D’Souza, Brady, Haensel, and D’Souza (2020) reported that seven- to nine-month-old bilingual infants shifted to a new stimulus more rapidly than monolinguals and switched more frequently between two visual stimuli than monolinguals. While the present study was not designed to test differences between monolinguals’ and bilinguals’ visual disengagement, there was no evidence to indicate group differences in visual shifting. Bilinguals took longer on average than monolinguals to visit the non-face areas, meaning that they did not scan the visual scene more rapidly overall than did monolinguals. There were also no differences in the average number of areas on the array that monolinguals and bilinguals visited (see Figure S7). It could be that previous findings reporting group differences in visual shifting are either specific to the first year of life and/or to certain contexts (Comishen et al., 2019; D’Souza et al., 2020; Kovács & Mehler, 2009; see also Dal Ben et al., 2022). Previous findings have been reported on tasks explicitly designed to elicit visual disengagement and shifting (Kalashnikova et al., 2020), so it may simply be that these effects do not generalise to the exploration of complex arrays with social and non-social areas (the task reported here). However, it could be that bilinguals’ increased attention to static faces is related to differences in attention to faces during naturalistic interaction, such as during episodes of joint attention. Joint attention likely facilitates the development of theory of mind (Tomasello, 2018), and research has indeed suggested that theory of mind may be stronger in bilinguals than in monolinguals (for meta-analysis, see Schroeder, 2018). Future research should examine the links between early language experience and visual attention in both lab-based and naturalistic environments to clarify these relationships and their potential cascading effects on young bilinguals’ developing social cognition.

## Attention maintenance

The second pre-registered hypothesis of this study was that bilinguals would show stronger attention maintenance to faces compared to monolinguals (https://osf.io/92tsa; Hypothesis 2) as measured by number of fixations to the face area. The results did not support this hypothesis. The previously reported effect of bilinguals’ increased attention maintenance to face over nonface areas compared to monolinguals reported in younger samples (Mercure et al., 2018) is not apparent in toddlers aged 15- to 18-months. One potential explanation for this seemingly developmental difference is that young bilinguals maintain their attention to the face area in case it starts to speak. In contrast, older bilinguals, after initial fixation, do not maintain their attention to a static face as they are aware it is unlikely to provide any visual articulation information that could be useful for speech perception.

The existing literature suggests longer sustained attention, which is distinct from but related to the constructs of attention capture and maintenance measured here, to faces in the face pop-out task may be linked to developmental difficulties (de Klerk et al., 2014; Elsabbagh et al., 2013). Elsabbagh et al. (2013) reported that 14-month-olds at familial risk of developing autism spectrum disorder spent more time looking to the face areas than did the control groups. de Klerk et al. (2014) found that, among infants at high risk of developing ASD, those who spent more time looking to face areas at seven months showed worse face recognition skills at three years than those who spent less time looking at face areas at seven months. There were no language group differences in the present sample of 15- to 18-month-olds’ overall duration of looking to faces, a null difference also reported in Mercure et al.’s (2018) four- to 10-month-old sample. It seems most likely that young bilinguals immersed in an intense period of phonological learning (four to eight months; Mercure et al., 2018) may maintain their attention to faces over the course of face pop-out, but that their increased attention maintenance to faces lessens towards the end of the first year and does not re-emerge in the second year. According to this view, bilinguals’ increased attention maintenance to faces is a transitional stage rather than an experience-related effect which endures throughout the first two years. Whether it has any beneficial consequences is unclear.

In summary, the data from the current study, in combination with previous data from our group, suggest that early exposure to bilingual language environments is linked to both transitory and sustained differences in visual attention for social stimuli. Bilingual infants’ increased attention capture by faces compared to monolinguals emerges in the second half of the first year (Mercure et al., 2018) and persists into the 15- to 18-month age window of the current study. However, bilingual infants’ increased attention maintenance to faces compared to monolinguals is a specific developmental phase characteristic of the first half of the first year (Mercure et al., 2018) that is not present in the older toddlers included here. Overall, early bilingualism is linked to robust differences in the speed at which toddlers automatically look towards faces in the first (Mercure et al., 2018) and the second (current study) year of life.

The effects of bilingualism identified here emerged despite wide-ranging variability in the bilingual groups’ non-English language–some with high degree of phonological overlap with English such as Dutch and some with low degree of phonological overlap such as Greek. Patterns of language mixing, home and nursery language environments, and degree of exposure to both languages were not controlled. Even across the wide-ranging variability inherent to bilinguals’ early language experiences, learning two spoken languages was related to faster automatic orientation to faces than non-faces. If it is the case that bilinguals capitalise on social-communicative cues available on the face, the results of the present study suggest bilingual effects on face processing generalise to non-moving, non-talking face stimuli.

## Conclusion

This study sheds new light on the intricacies of universal and experience-sensitive developments in toddlers’ processing of visual social information. As in studies with young infants in the first year of life (Elsabbagh et al., 2013; Gliga et al., 2009; Mercure et al., 2018), the 15- to 18-month-olds studied here looked faster and more frequently to social over non-social stimuli, regardless of the variability in their early language experiences. However, toddlers’ rapid visual orientation to social stimuli (over non-social stimuli) was sensitive to early language experience, such that bilinguals were faster to look to static faces and slower to look to non-faces in an array than monolinguals. It may be the case that early experience of two spoken languages rather than one is related to stronger immediate orientation to social over non-social visual information throughout the first two years of life. There were no group differences in attention maintenance, suggesting that early language experience is not linked to differences in the number of times 15- to 18-month-old monolinguals and bilinguals look to social over non-social stimuli. These results suggest that some of the attentional mechanisms underlying toddlers’ automatic looking to faces, which are present across age and developmental variability, are nonetheless sensitive to early language experience.

## Supplementary Material

For supplementary material accompanying this paper, visit https://doi.org/10.1017/S136672892200092X

Supplementary Material

## Figures and Tables

**Table 1:** Fixation latency and count measures to each area on face pop-out slides by group.

Area of Interest	Monolingual (*n* = 32)				Bilingual (*n* = 26)			
	Latency		Count		Latency		Count	
	M (SD)	Range	M (SD)	Range	M (SD)	Range	M (SD)	Range
Face	1.32 (0.69)	0.53 – 3.17	2.99 (0.85)	1.25 — 4.50	1.00 (0.54)	0.43 — 2.68	3.11 (0.86)	1.00 — 4.86
Noisy Face	2.23 (0.70)	1.17 — 4.05	1.88 (0.60)	0.67 — 3.25	2.50 (0.86)	1.15 — 4.17	1.91 (0.60)	0.62 — 2.88
Bird	2.63 (1.17)	0.50 — 4.84	1.99 (0.94)	0.62 — 4.25	2.37 (1.04)	1.02 — 5.02	2.07 (0.97)	0.00 — 4.12
Car	2.17 (1.01)	0.85 — 5.33	2.37 (0.95)	0.88 — 5.75	3.39 (1.29)	0.84 — 6.40	1.75 (1.02)	0.50 — 4.29
Phone	2.63 (1.17)	0.50 — 4.84	1.99 (0.94)	0.62 — 4.25	2.37 (1.04)	1.02 — 5.02	2.07 (0.97)	0.00 — 4.12

*Note*. Latency is calculated as the time between onset of the face pop-out slide and toddlers’ first fixation to the area of interest. Latency units are seconds. Count is calculated as the number of fixations to the area of interest. Both measures were averaged within-participant across trials to standardise for number of trials completed.

## Data Availability

The datasets generated during the current study are not publicly available due to the highly sensitive nature of children’s private data but are available from the corresponding author on reasonable request.
