# Supplementary material for: Bilingual toddlers show increased attention capture by static faces compared to monolinguals
Source: Biling (Camb Engl). Author manuscript; Available in PMC 2023 Aug 26. (PMC7614981; doi:10.1017/S136672892200092X)
Supplement: Supplementary Material [file EMS158135-supplement-Supplementary_Material.pdf]

Supplementary Materials

Figure S4. *Boxplot of pre- and post-COVID samples' fixation latencies to face and non-face areas of interest.*

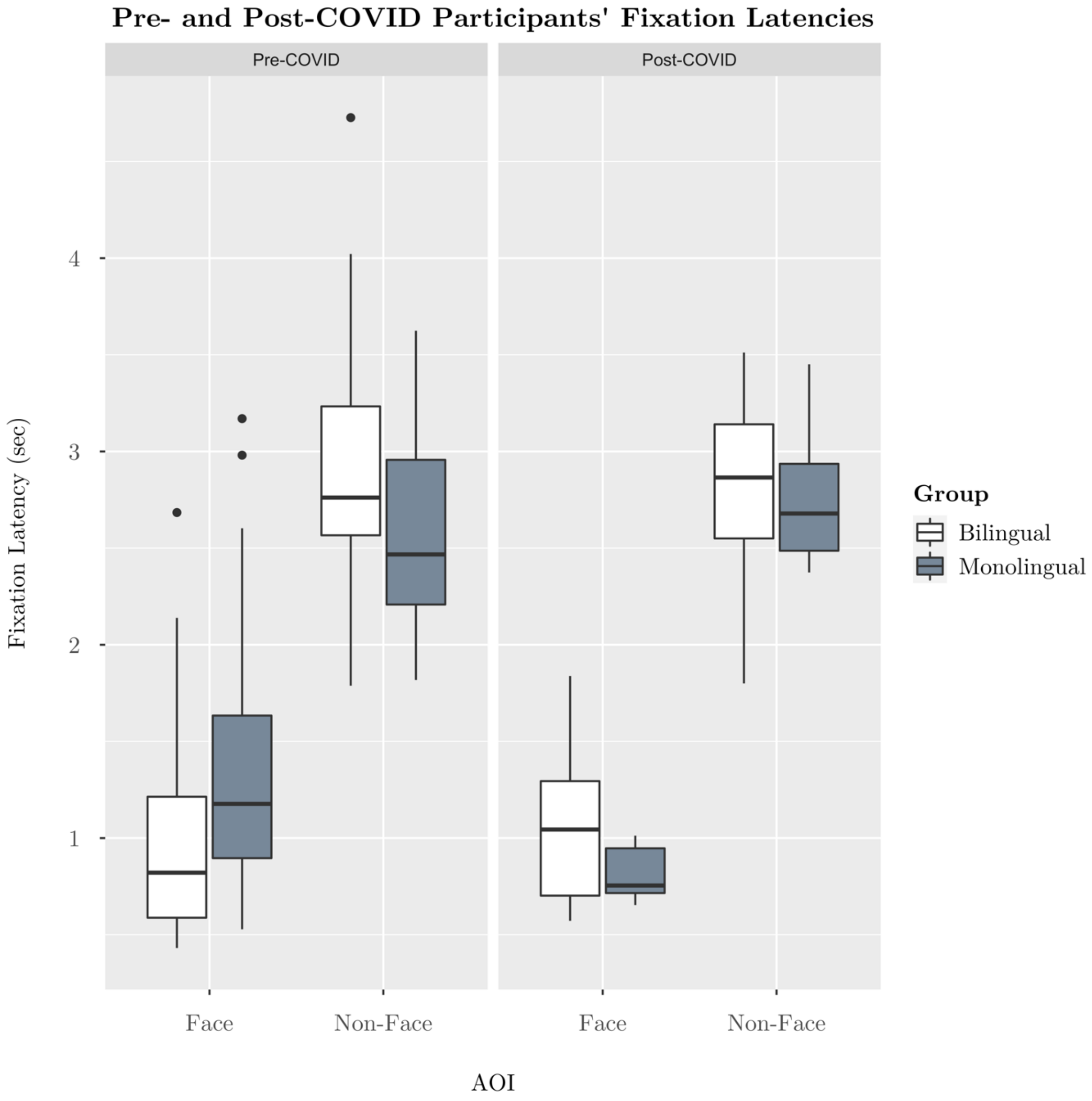

Note. The pre-COVID sample (monolinguals: *n* = 32, bilinguals: *n* = 26) shown on the left-hand panel and post-COVID monolinguals (*n* = 5) and bilinguals (*n* = 8) are shown on the righthand panel. Bilinguals are represented in white, and monolinguals are represented in grey. Fixation latency is calculated as the time between onset of the face pop-out slide and toddlers' first fixation to the area of interest (face or non-face). The non-face values were calculated within-participant as an average of toddlers' fixation latencies to the four non-face areas (i.e., noisy face, car, phone, and bird). The error bars represent standard error.

Figure S5. *Boxplot of average overall fixation duration on face vs non-face areas per group.*

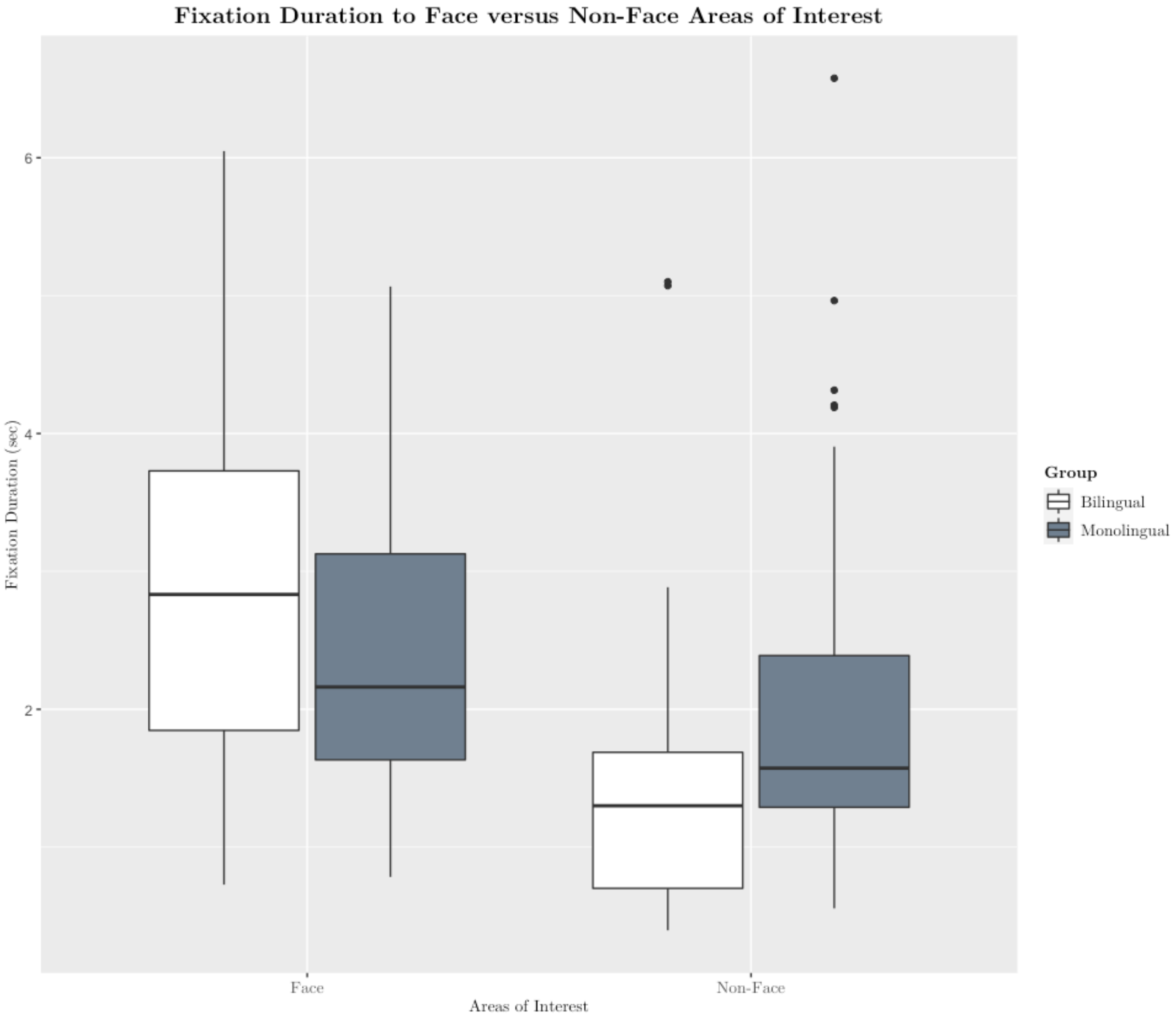

Note. The average duration of looking to the face area was averaged within-participant to standardise for number of face pop-out trials completed. Each face pop-out trial contained an array with five areas of interest: face, noisy face, car, phone, and bird. Bilinguals are represented in white, and monolinguals are represented in grey. The error bars represent standard error.

Figure S6. *Boxplot of average proportion fixation duration on face out of all looking time to the screen per group.*

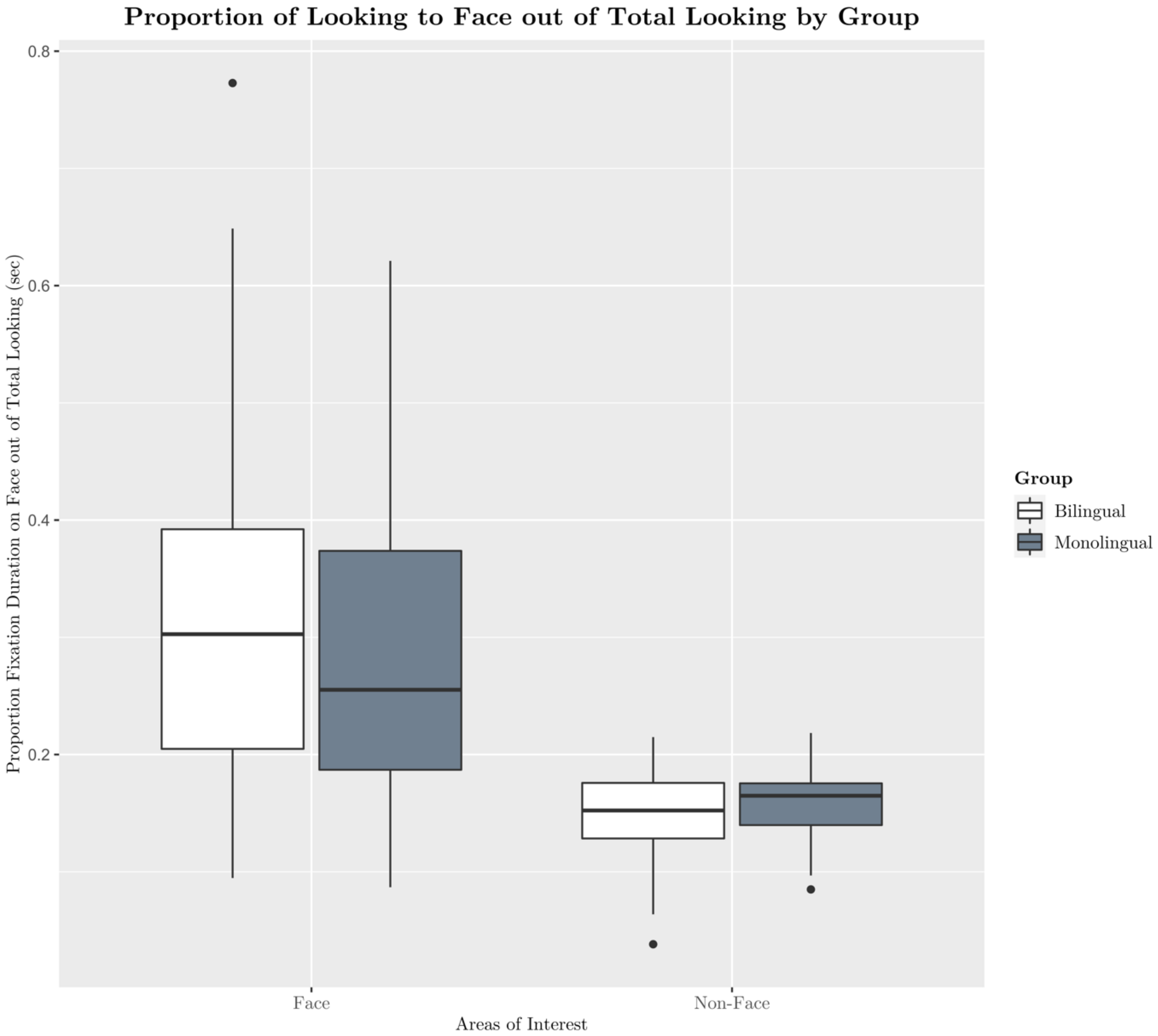

Note. The average duration of looking to the face area was averaged within-participant to standardise for number of face pop-out trials completed and divided by participants' total looking time across all trials completed. Each face pop-out trial contained an array with five areas of interest: face, noisy face, car, phone, and bird. Bilinguals are represented in white, and monolinguals are represented in grey. The error bars represent standard error.

Figure S7. *Boxplot of number of areas of interest visited on per group.*

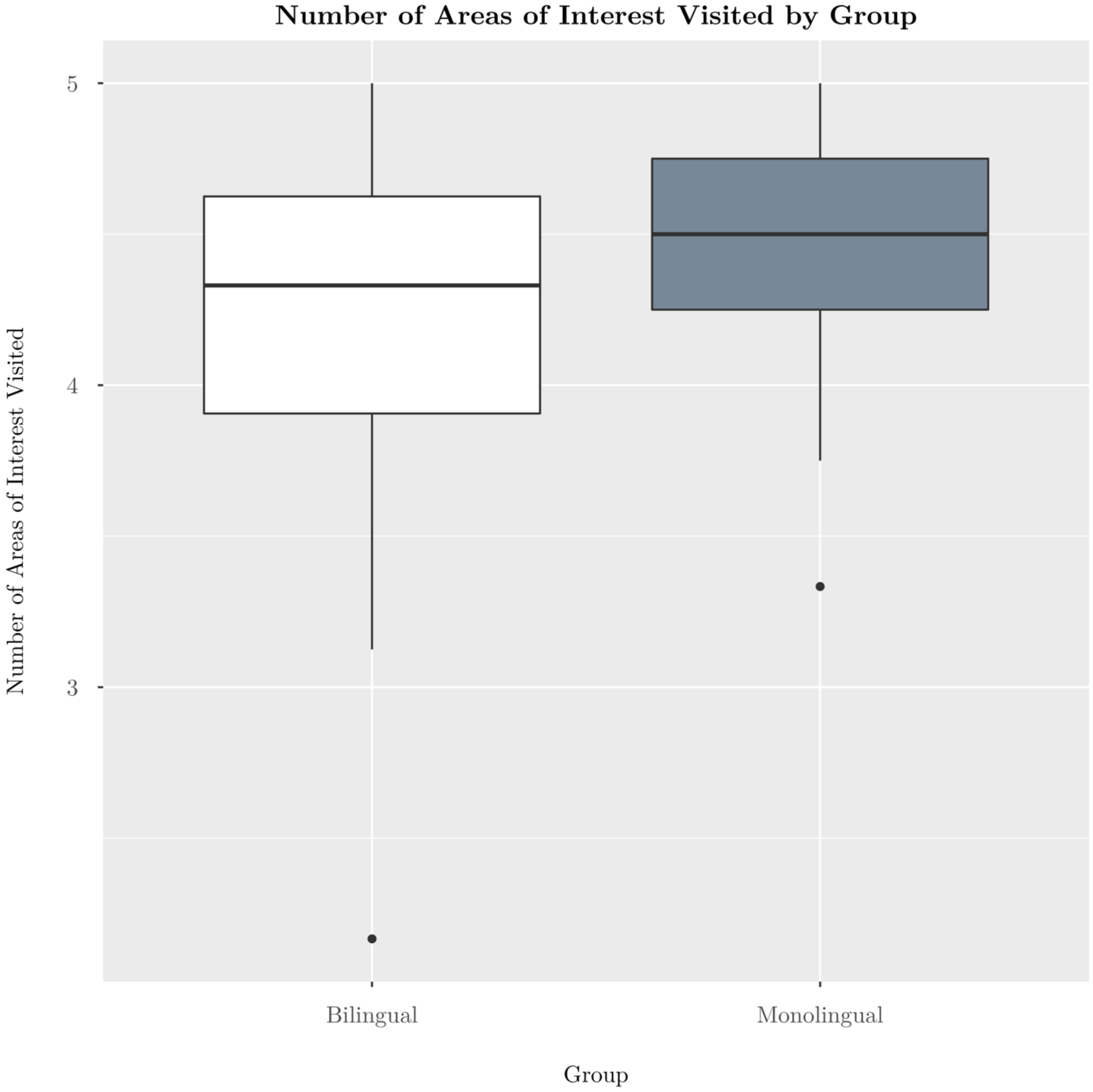

Note. The number of areas of interest visited were averaged within-participant to standardise for number of face pop-out trials completed. Each face pop-out trial contained an array with five areas of interest: face, noisy face, car, phone, and bird. A visit to areas of interest was defined as at least one fixation to the area. Bilinguals are represented in white, and monolinguals are represented in grey. The error bars represent standard error.
